# What motivates customers to repurchase online under social distancing?

**DOI:** 10.3389/fpsyg.2023.1155302

**Published:** 2023-07-25

**Authors:** Weishen Wu, Gumilang Widiatmo, Dalianus Riantama

**Affiliations:** ^1^Department of Information Management, Da-Yeh University, Changhua, Taiwan; ^2^College of Management, Da-Yeh University, Changhua, Taiwan; ^3^Management Department, BINUS Business School Undergraduate Program, Bina Nusantara University, Jakarta, Indonesia

**Keywords:** online repurchase intention, social contingency, performance confirmation, effort confirmation, customer perceived value, habit

## Abstract

Global e-commerce is growing rapidly during the COVID pandemic. Previous research on customers' online shopping decisions rarely considered social distancing. To investigate customers' continued intention toward online purchases while socially isolated, we propose a framework based on the UTAUT model. A survey of 330 valid samples was collected through an online survey among internet users during a period of social distancing in Indonesia. Hypotheses were validated using a structural equation modeling approach. The results showed that social contingency is the most influential factor on customers' intention to repurchase online under social restriction conditions, followed by customer perceived value and other significant factors. The findings contribute to providing a new understanding of customers' online repurchase intentions when they are in a contingency situation.

## Introduction

The growth of e-commerce has brought about significant modifications in personal online buying behavior. The advantage of e-commerce over traditional retail is that it eliminates geographical boundaries at any time (Mohanty et al., 2007). Since 2019, the COVID pandemic triggered tremendous growth in online sales as physical store visits were restricted, leading consumers to shift to online shopping (Dannenberg et al., 2020). Governments worldwide have implemented social distancing policies in response to the COVID outbreak to curb its spread. Social distancing measures aim to increase physical space between individuals to reduce the risk of virus transmission (Gross and Padilla, 2020). While this policy has restricted brick-and-mortar businesses, it has accelerated the growth of e-commerce (Fahrizal, 2020). This evolution has resulted in the maturation of online shopping as a retail channel and significant changes in people's purchasing habits (Le et al., 2022). Social distancing has emerged as the new norm that affects consumer purchasing behavior globally (Wang et al., 2021). According to Von Abrams (2021), total global online retail sales reached 17.9% in 2020 and rose to 20.3% in 2022.

Many governments implemented a social distancing policy which limited people's activities in public areas and facilities (Winanti et al., 2022). Consequently, workplaces have implemented work-from-home (WFH) measures to comply with social distancing policies, including in Indonesia. As Indonesian consumers practice social distancing to avoid the risk of COVID infection, online shopping has become increasingly popular in Indonesia (Adam et al., 2022). The rise of online and digital transactions (Prasetya et al., 2021) has led to a 25–30% increase in e-commerce in Indonesia during the pandemic (Pink, 2021).

Research conducted prior to the COVID pandemic using the Unified Theory of Acceptance and Use of Technology (UTAUT) revealed several factors that influenced customers' intention to shop online, such as performance expectations, effort expectations, social influence, and facilitating conditions (Venkatesh et al., 2003), as well as hedonic motivation, price value, and habit (Venkatesh et al., 2012). However, studies conducted during the early stages of the pandemic found that effort expectancy (Kadir and Ismail, 2022) and social influence (Erjavec and Manfreda, 2022) did not significantly impact customers' online shopping intentions. Recent research conducted during the pandemic suggests that major factors influencing customers' online purchasing decisions include performance expectancy (Zhao and Bacao, 2021), effort expectancy (Musyaffi, 2021), habit (Sheikh et al., 2017), and customer perceived value (Asti et al., 2021). These four UTAUT constructs were frequently identified in studies conducted both before and at the beginning of the COVID-19 outbreak.

The impact of social distancing on customers' online repurchase decisions has become an important concern, as highlighted by Maulidina et al. (2020). Given that social distancing measures affect the external environment rather than situational factors (Gehrt and Yan, 2004), the factors that influence customers' online repurchasing may differ from previous findings. However, recent research on consumer online repurchase has largely overlooked the context of social distancing. This study aims to address this gap by introducing a new variable, social contingency, as an external factor that can change customers' decisions to repurchase items online. As social distancing policy is crucial in mitigating the spread of epidemic diseases, it is important to gain a better understanding of the contextual factors that influence customers' online repurchasing decisions when socially isolated.

This study intends to investigate consumer online repurchase intentions during the pandemic, with a specific focus on developing countries, in addition to the UTAUT model. Prior research on online shopping has primarily concentrated on initial purchase intentions and paid less attention to frequent online shoppers or experienced customers. Therefore, this study aims to bridge this gap by applying the Expectation Confirmation Theory (ECT), a cognitive theory that explains post-purchase behavior, including repurchase intentions. ECT proposes that consumers repurchase intentions for a product or service are significantly influenced by their prior experience with it. By incorporating ECT, this study seeks to provide authentic factors that affect consumer repurchase intentions during the pandemic.

ECT emphasizes the importance of the confirmation construct. Hence, in this study, we integrate the UTAUT2 constructs (i.e., performance expectancy and effort expectancy) with the confirmation construct to create performance and effort confirmation. This integration attempts to enhance theoretical contributions to post-purchase consumer behavior in online shopping. The variables used in this study are performance confirmation, effort confirmation, customer perceived value, social contingency, and habit toward online shopping continuance intention. Although ECT provides a comprehensive theoretical framework, it remains unclear how social distancing impacts customers' online repurchase intentions. Additionally, theoretical models that consider social distancing and customer online repurchase intentions during the pandemic are scarce, particularly in e-commerce growing countries such as Indonesia. Therefore, this study seeks to explore the effects of social distancing on customers' online repurchase intentions during the pandemic. Two research questions are raised as below:

RQ1: What factors influence customers' continuous intentions toward online shopping during social distancing?
RQ2: What are the relationships among these factors that influence customers' intentions to continue online shopping?

This study holds significant importance as it introduces a model to measure customers' online shopping intentions under social contingencies like social distancing. Findings offer novel insights for online shopping research and practice in the post-COVID era. In addition to the core factors influencing customers' continuous online shopping intentions derived from UTAUT models, this study integrates a contextual variable called social contingency. The forthcoming sections present a literature review, hypothesis development, a methodology for validating the hypotheses, the results and implications, and the conclusion.

## Literature review

### The UTAUT models

The Unified Theory of Acceptance and Use of Technology (UTAUT) was developed by Venkatesh et al. (2003). It is an integrated model based on socio-cognitive perspectives on users' initial acceptance of information technology. The original UTAUT model, also known as UTAUT1, proposes four constructs that affect users' intentions to use a specific technological product or service, namely performance expectancy, effort expectancy, social influence, and facilitating conditions. UTAUT1 has a 70% explained variance in behaviors, which outperforms other behavioral intention models (Venkatesh et al., 2003; Lu et al., 2009). Many studies have found that the four constructs of UTAUT1 can explain 71%−75% of the total variances influencing consumers' online purchase intentions (Adnan et al., 2018; Wijaya et al., 2022).

The original UTAUT model was later expanded with the inclusion of three additional constructs, namely hedonic motivation, price value, and habit, namely UTAUT2. This modification aimed to enhance its ability to predict consumer behavior. UTAUT2 demonstrated a significant improvement in its predictive power for consumption intentions compared to UTAUT1, with seven of the total UTAUT2 constructs contributing to an increase in the explained variance from 56% to 74% (Venkatesh et al., 2012). Table 1 provides the definitions of the core constructs in both UTAUT models.

**Table 1:** Construct definition of UTAUT models.

	**Constructs**	**Definition**	**Source**
UTAUT1	Performance expectancy	Individual's belief that using technology can improve working or living outcomes	Venkatesh et al. (2003)
	Effort expectancy	Individual's perceived ease of using technology	
	Social influence	The extent to which an individual perceives that other people (such as family and friends) think that he or she should utilize a particular a new technology	
	Facilitating conditions	The extent to which an individual believes that an organizational and technical infrastructure that exists to promote the use of a system	
Added by UTAUT2	Hedonic motivation	Pleasure or happiness enjoyed from using the technology	Venkatesh et al. (2012)
	Price value	The consumers' cognitive trade-off between the perceived benefits and the monetary expenses of a consumption	
	Habit	The degree to which consumers tend to use technologies or technological products automatically because of their learning experience	

Compared to other user behavioral models such as the theory of reasoned action (TRA), the theory of planned behavior (TPB), and the technology acceptance model (TAM), the UTAUT models show higher explanatory power in predicting customers' initial purchase intentions (Al-Qeisi et al., 2015; Taherdoost, 2018; Liu et al., 2022). This is because the UTAUT models integrate the above models into a unified framework (Venkatesh et al., 2003). Several studies have demonstrated that the UTAUT model is more effective in explaining consumer behavior related to technology acceptance compared to other models (Lin et al., 2019; Alghazi et al., 2021; Andrews et al., 2021; Bu et al., 2021), indicating its robustness. When compared to the previously mentioned theories, which explained only 17% to 42% of the variance in usage intention and behavior related to technology, the UTAUT model stands out with its high explanatory power of 70% in evaluating technology acceptance (Chao, 2019). Nevertheless, the efficacy of UTAUT constructs in predicting customer online repurchase intentions under social distancing remains unclear.

### UTAUT factors influencing customers' online repurchase intention

There is a significant difference when comparing consumer online shopping behaviors before and during the COVID-19 pandemic. Before COVID, more customers went shopping in person even though it was not preferred (Bridges and Fowler, 2022). In contrast, social distancing policies and consumers' worries about shopping in crowded places have increased interests in online shopping (Ellison et al., 2020). Consumers have been abruptly compelled to alter their priorities for online shopping because of COVID-19 (Pan et al., 2020). During the pandemic, customers found that online shopping was convenient, affordable, and helps them overcome the stress brought on by new sanitary standards and restrictions at retail shops (Guthrie et al., 2021). Besides predicting consumers' initial acceptance, the UTAUT models have often used to investigate customers' online repurchase intentions. Thus, this study investigates the major influential factors influencing customers' online repurchase intentions based on the UTAUT model before and during COVID.

Prior to the COVID-19 pandemic, studies in e-commerce have identified several factors from the UTAUT models that significantly impact customers' online repurchase intentions. Tam et al. (2020) found that habit, performance expectancy, and effort expectancy were critical drivers of customers' intentions to purchase online using mobile payment. Lee et al. (2019) and Alalwan (2020) identified habit and performance expectation as significant factors in customers' continued intentions to use online food ordering services. Zhou (2011) indicated that performance expectancy, social influence, and facilitating conditions impacted customers' continued intention to use mobile shopping and payment services. Similarly, Purohit et al. (2022) found that effort expectancy and facilitating conditions significantly influenced customers' online repurchasing decisions. Additionally, perceived value significantly affected customers' repurchasing intentions in m-commerce (Marinković et al., 2019) and mobile payment services (Oh and Kim, 2020) in a similar context.

During the COVID pandemic, studies have utilized the UTAUT models to examine customers' continuous shopping intentions with m-commerce. The significant influential factors identified during this period include performance expectancy and effort expectancy (Ramos, 2022), social influence (Zhao and Bacao, 2020; Muangmee et al., 2021), facilitating conditions and hedonic motivations (Shoheib and Abu-Shanab, 2022; Vinerean et al., 2022), habit (Nam and An, 2021), and perceived value (Nguyen et al., 2022). In contrast to findings prior to and during COVID, the most commonly used constructs in UTAUT models for predicting customers' online shopping intentions include performance expectancy, effort expectancy, habit, and perceived value. The constructs of UTAUT1 and UTAUT2 are listed in Table 2.

**Table 2:** Summary table of UTAUT1 and UTAUT2.

**Study**	**Application**	**Model**	**Country**	**PE**	**EE**	**SI**	**FC**	**HM**	**Habit**	**Perceived value**
Kim and Yoo (2020)	Mobile payment	UTAUT2	Korea	S	NS	S	NS	S		-
Tam et al. (2020)	Mobile apps	UTAUT2	Portugal	S	S	NS	NS	NS	S	-
Lee et al. (2019)	Food delivery apps	UTAUT2	Korea	S	NS	S	NS	NS	S	-
Zhou (2011)	Mobile payment	UTAUT	China	S	NS	S	S	-	-	-
Purohit et al. (2022)		UTAUT	India	NS	S	NS	S	-	-	-
Alalwan (2020)	Mobile food ordering apps	UTAUT2	Jordan	S	NS	NS	NS	S	S	-
Marinković et al. (2019)	m-commerce	UTAUT	Serbia	NS	NS	NS	-	-	-	S
Oh and Kim (2020)	m-payment	UTAUT	Korea	-	-	NS	NS	-	-	S
Ramos (2022)	Food delivery apps	UTAUT2	Mexico	S	S	-	-	-	-	-
Muangmee et al. (2021)	Food delivery apps	UTAUT	Thailand	S	S	S	-	-	-	-
Zhao and Bacao (2020)	Food delivery apps	UTAUT	China	S	NS	S	-	-	-	-
Shoheib and Abu-Shanab (2022)	Social commerce	UTAUT2	Qatar	NS	NS	-	S	S	NS	-
Vinerean et al. (2022)	m-commerce	UTAUT2	Romania	S	-	S	-	S	-	-
Nam and An (2021)	Food delivery apps	UTAUT2	Vietnam	S	S	S	S	S	S	-
Nguyen et al. (2022)	Food delivery service	UTAUT2	Vietnam	-	-	-	-	-	S	S

Note. “S” = significant, “NS” = non-significant, “-” = relation not examined.

While performance expectancy and effort expectancy are significant predictors of customers' initial purchase intentions, they may not be as relevant to customers' post-purchase decisions as they are to customers who have experience shopping online.

### Performance confirmation and effort confirmation

The formation of customer expectations and confirmation occurs at different stages of the purchasing process. Prior to making a purchase or during the initial use of a product or service, a customer's belief about the potential utility to be gained is referred to as expectation (Oliver, 1980). According to the expectation-confirmation theory, consumers evaluate a product's performance not only based on their initial expectations but also on performance confirmation. Confirmation refers to the consumer's perspective of the alignment between their use expectations and actual current results (Bhattacherjee, 2001). In this study, we integrate “confirmation” with the UTAUT constructs by defining performance confirmation as a consumer's perception of the alignment between online shopping expectations and actual outcomes, and effort confirmation as a consumer's perception of the alignment between expected online transactions and actual operations.

Hsu and Lin (2015) found confirmation was positively correlated with customer perceived value in mobile app usage and significantly impact on users' intention to purchase mobile apps. The effect of confirmation on customers' perceived value has been shown to influence consumers' continuous intention to use accommodation applications significantly (Kim et al., 2019). Customers are more likely to increase their perceived value of a product/service when its performance and effort are confirmed. Confirmation of performance and effort of online shopping experiences can be attributed to customer perceived value. Based on the above discussion, we argue that performance confirmation and effort confirmation are appropriately used as antecedents of CPV, replacing performance expectancy and effort expectancy in the UTAUT1 model in the context of consumers' online repurchase intention. Hence, two hypotheses are postulated as follows:

H1: Performance confirmation has a positive effect on customer perceived value.
H2: Effort confirmation has a positive effect on customer perceived value.

### Customer perceived value

Perceived value, according to Zeithaml (1988) and Dodds et al. (1991) definition, is the overall evaluation of a product's or service's usefulness by consumers, based on their perceptions of what they receive and what they give, reflecting the trade-off between perceived benefit and perceived risk. Chae et al. (2020) suggest that perceived value is the customer's assessment of the product's utility, as well as their perception of personal effort and benefit. Similarly, Miao et al. (2022) defines perceived value as consumers' opinions of goods and services. Perceived value plays a critical role in the exchange transaction (Dhingra et al., 2020), as it is the point at which customers perceive that the benefits, they receive outweigh the price they pay (Sinha and Verma, 2020). Known as customer perceived value (CPV), Kotler and Keller (2006) explain that it is the gap between a potential customer's evaluation of all the benefits and drawbacks of a product or service compared to other options. Customers gauge the worth of the benefits they received in relation to the money they paid and are willing to pay more if they feel they are getting better value for their money.

The UTAUT2 model defines price value as the balance between the perceived advantages of using an application and its monetary cost, according to Venkatesh et al. (2012). In contrast, customer perceived value (CPV), as explained by Aini et al. (2019), incorporates not only benefits and costs but also the evaluation process that customers undergo. Gan and Wang (2017) explored CPV by considering consumers' perceptions of utilitarian, hedonic, and social benefits, as well as perceived risk. Meanwhile, Lin et al. (2018) expanded CPV to include consumer perceived usefulness, perceived ease of use, and perceived risk, which encompass economic and privacy risks. Their findings indicate that these factors significantly influence customers' intentions to continue purchasing online.

This study defines CPV as a customer's perception of the benefits and trade-offs associated with obtaining more tailored products or services that provide greater value for money (Parasuraman and Grewal, 2000). Previous research has shown that CPV is a key driver of online repurchasing behavior or intentions, as noted by Ali and Bhasin (2019) and Ijaz and Rhee (2018). Moreover, perceived value is an essential precursor to repurchasing intention, according to Gligor and Bozkurt (2020). As such, it is vital to sustain CPV to foster long-term customer relationships and encourage repurchasing intention, as outlined by Kim et al. (2012). Perceived value is a major consideration in online purchasing decisions (Chiu et al., 2014). CPV can be increased if e-commerce stores offer personalized goods or services to fit customers' individual needs (Jiang et al., 2015). When a product or service bought from the Internet provides a higher perceived value, customers will be more likely to repurchase it (Guo and Li, 2022). Past studies showed when customers receive a good deal for money from online shopping, they will likely purchase it in the future.

Customer perceived value (CPV) encompasses the efforts, costs, and risks that customers undertake when deciding to make repeat purchases. Previous studies have demonstrated that CPV positively impacts an individual's intention to repurchase networking services (Kuo et al., 2009), participate in repeat online group buying (Hsu et al., 2015), or revisit online stores (Fang et al., 2016). Furthermore, customer perceived value is a significant factor influencing online shopping repurchase intention (Yang and Lee, 2010; Pobee, 2021), and it plays a crucial role in enhancing online shopping continuance during the COVID-19 pandemic (Prasetyo et al., 2021). As a result, customers are more likely to engage in repeat online purchases once they perceive greater value from their previous online shopping experiences.

If customers perceive a high level of customer perceived value (CPV) from an online retail store, they are more likely to engage in repeat purchases from that store (Irshad, 2016). CPV has a significant impact on online shoppers' repurchase intentions, as noted by Wu et al. (2014). Furthermore, the more a consumer perceives the value of an online shopping experience, the more likely they are to reinforce the habit of shopping online (Aarts and Dijksterhuis, 2000). As customers derive more value from technology, such as online shopping during the pandemic, their online shopping habits are likely to become more entrenched (Nguyen et al., 2022). In the context of online shopping, CPV has a positive correlation with online shopping habits, as found by Chiu et al. (2012). Customers who frequently engage in online purchases are more susceptible to the influence of perceived value (Cheng et al., 2017). Therefore, the relationship among CPV, habit, and online shopping continuance intention can be postulated as follows:

H3: Customer perceived value has a positive effect on online shopping continuance intention.
H4: Customer perceived value has a positive effect on habit.

### Social contingency

According to Chou and Hsu (2016), consumers' purchasing decisions are influenced by their circumstances and cognitive elaboration. When the COVID pandemic occurred, consumers became worried about supply shortages and price increases, leading to panic buying of daily necessities (Yoon et al., 2018; Wang et al., 2020). Social distancing is a new external environmental factor that can be classified as a large-scale social contingent occurrence, unlike situational factors that are unique to a particular time and place and do not stem from knowledge of personal stimuli that affect current behavior systematically (Belk, 1975). Due to social distancing restrictions, people may find themselves in social isolation, which refers to being physically and chronologically alone during emergencies (Al Amin et al., 2022).

This study introduces the term “social contingency” as a variable to describe how unexpected social environments can influence individual behaviors. Social distancing measures, such as lockdowns, social isolation, and restrictions due to COVID pandemic, are a prime example of social contingency. Therefore, social contingency is defined as external environmental factors that can quickly change people's behaviors, such as panic buying, remote work, distance learning, and others.

Vinerean et al. (2022) have reported that consumers have significantly adopted and depended on online shopping directly from e-stores or online shops during COVID lockdowns and restrictions that were characterized by confinement. Consequently, there has been a surge in online shopping due to the government's social distancing policies that imposed lockdowns and strict protocols during the pandemic (Gruntkowski and Martinez, 2022). The implementation of social distancing measures to curb COVID-19 transmission has influenced consumers' decisions to shift to contactless channels such as cashless payments, leading to a significant increase in home delivery and online shopping (Pantano et al., 2020; Wang et al., 2021; Alaimo et al., 2022).

The pandemic-induced social distancing measures have not only affected consumer behavior but have also accelerated the growth of e-commerce and online shopping (Ivascu et al., 2022). Recent studies suggest that social distancing has not only led to an increase in consumers' intentions to purchase online (Itani and Hollebeek, 2020) but also strengthened their commitment to do so (Nguyen et al., 2020; Toska et al., 2022). The concept of social contingency can be applied to consumer behavior as well as other external situations, such as people's responses to financial crises, conflicts between nations, and extreme weather conditions.

Social distancing has compelled consumers to switch from physical brick-and-mortar stores to online stores for their shopping needs (Reddy, 2020). These shifts have not only intensified customers' online shopping habits (Mejía-Trejo, 2021) but have also increased their intentions to repurchase online (Gefen and David, 2000). As a result, social distancing has discouraged people from visiting physical stores while encouraging them to shop online. Therefore, relationships among social contingency, habit, and online shopping continuance intention can be hypothesized as follows:

H5: Social contingency has a positive effect on habit.
H6: Social contingency has a positive effect on online shopping continuance intention.

### Habit and online shopping continuance intention

As per previous literature, a habit can be described as an automatic behavioral inclination that an individual acquires over time (Limayem and Hirt, 2003). Habits are automatic responses that are constrained by circumstances or stimuli (Aarts et al., 1998). A person's psychological tendency to repeat a behavior is referred to as a habit (Neal et al., 2012). Habits are actions that become natural, and individuals are typically unaware of these patterns. Individuals who have a habit of online shopping will continue to make purchases, whether necessary or not (Ghias et al., 2022). Therefore, in this study, a habit is defined as a customer's routine of purchasing goods or services from online stores. Habit has been used to describe the development of customers' perceptions and consumer behavior, such as continued online shopping, in both online and offline shopping (Honkanen et al., 2005).

According to Liao et al. (2006), when using a website becomes habitual, it can reinforce the intention to continue using the internet for activities such as online shopping. The COVID pandemic led to more consumers switching to the Internet, resulting in increased spending on online shopping (McKinsey, 2020). This, in turn, reinforces customers' online purchasing habits (Tao et al., 2022) and strengthens the influence of their purchase intention (Chen et al., 2022). Nguyen et al. (2022) discovered that habit plays a crucial role in driving customers' unconscious response to continue shopping online, and it is a potent influence on their intention to keep shopping online.

During the COVID pandemic, people have gradually relied on shopping online (Rui, 2022). Lavuri (2021) and Gefen (2003) observed this habit directly influences consumers' intentions to shop online. In the context of online shopping, customer habits have a positive correlation with repurchase intentions (Khalifa and Liu, 2007; Huang and Chen, 2017) because the creation of online shopping habits plays a crucial role in determining customers' repurchase intentions (Nazir et al., 2023). Shoppers who have established online shopping habits are more likely to repurchase and enhance their online repurchase intentions (Anderson and Sullivan, 1993; Lin and Lekhawipat, 2014). Consequently, it can be inferred that the frequency with which buyers engage in online shopping directly impacts their intention to online repurchasing (Mohamed et al., 2014). Therefore, the relationship between habit and online shopping continuance intention is hypothesized as follows:

H7: Habit has a positive effect on online shopping continuance intention.

**Figure 1:**
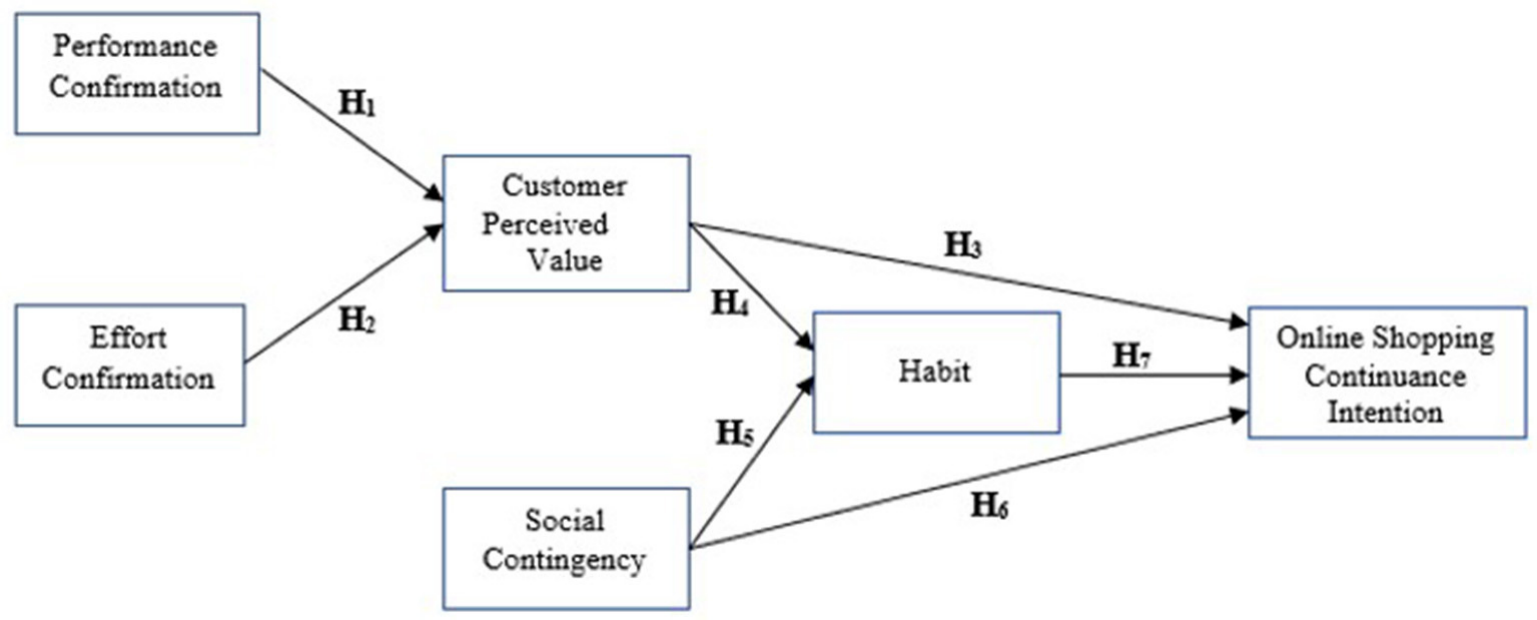
Research framework.

Based on literature prior to and during the COVID pandemic, it was found that the four primary factors of UTAUT—performance expectancy, effort expectancy, perceived value, and habit—were frequently utilized in studies of customers' online repurchasing behavior. However, these factors may not align with customers' current considerations when it comes to online repurchasing. It is hypothesized that an individual's intention to repeat an action is primarily influenced by their previous actions and their inner assessment and reaction to external circumstances, which triggers a behavioral intention. Thus, habit is viewed as a sustainable driver for online repurchases when online shopping becomes a routine for customers. Customer perceived value (CPV) is used due to its consolidated nature and its influence on habit and online repurchase intention. In addition, a new external variable called social contingency is introduced to stimulate habit and intention to continue shopping online. Performance confirmation and effort confirmation are identified as antecedents of CPV, replacing performance expectancy and effort expectancy in UTAUT models. Therefore, based on the above assumptions, a research framework is presented in Figure 1.

### Operational definitions and measurements

The research framework includes six variables, with operational definitions for each variable listed in Table 3.

**Table 3:** Operational definitions of variables.

**Variable**	**Operational definition**	**Reference**
Online shopping continuance intention	The possibility of consumer repurchases on the Internet in near future	Mohamed et al. (2014)
Habit	The extent to which consumers tend to shop on the Internet automatically	Liao et al. (2006)
Performance confirmation	Consumer's perception of the congruence between online shopping expectations and their actual outcome	Bhattacherjee (2001)
Effort confirmation	Consumer's perception of the congruence between the expected online transactions and their actual operations	Bhattacherjee (2001)
Customer perceived value	Consumers' perceptions of net benefits obtained from online shopping in terms of a trade-off between advantages and sacrifices	Parasuraman and Grewal (2000)
Social contingency	A social state that affects individuals' behaviors	The present study

Measurements for each variable were chosen from previous studies and adjusted according to its operational definition, as shown in Table 4.

**Table 4:** Measurements of variables.

**Variable**	**Code**	**Measurement**	**Reference**
Online shopping continuance	OSCI1	I am thinking of repurchasing online soon	Mohamed et al. (2014)
Intention (OSCI)	OSCI2	I will make purchases again on the Internet	
	OSCI3	I plan on shopping from online stores another time	
	OSCI4	I am likely to buy stuff frequently on the Internet	
Habit (HA)	HA1	I often shop online for different types of products or services	Liao et al. (2006)
	HA2	I regularly purchase goods on the Internet	
	HA3	Searching for stuff trending on the Internet has become my routine	
	HA4	I get used to online purchases recently	
Performance confirmation (PC)	PC1	Delivery of online orders is fast to me	Bhattacherjee (2001); Venkatesh et al. (2003)
	PC2	I feel convenient to order goods from online stores	
	PC3	Using online shopping makes the handling of payments easier	
Effort confirmation (EC)	EC1	Online shopping can be done anywhere	Bhattacherjee (2001); Venkatesh et al. (2003)
	EC2	Shopping on the Internet saves my time	
	EC3	Steps of online transactions are simple to me	
	EC4	Shopping online makes my life easier	
Customer perceived value (CPV)	CPV1	I can obtain desired products or services from the Internet	Zeithaml (1988); Dodds et al. (1991)
	CPV2	Buying products or services online makes me feel better value for money	
	CPV3	Online stores provide me more personalized products or services	
Social contingency (SC)	SC1	I am worried about a short supply of daily necessities because of panic buying	Present study
	SC2	I have needs for living even social emergencies are occurred	
	SC3	I choose the best alternative for preventing risks	
	SC4	I have a sense of urgency in living while social distancing	
	SC5	I am anxious about life beyond my control during pandemic	

## Research methodology

The questionnaire was developed based on the relevant literature and includes demographic questions and various measurements. All measurements were measured with five-point Likert scales, ranging from 1 = “strongly disagree” to 5 =“strongly agree”. Since the respondents in this study are Indonesian consumers, the questionnaire items were translated into Indonesian language with proofreading. Next, to reduce language bias, a pilot study of bilingual questionnaires (English and Indonesian) was distributed to 30 participants who could read English. Based on the results, few items were revised to achieve the same meanings, removing response bias (Forza, 2002).

### Data collection

The e-commerce market in Southeast Asia has grown rapidly in recent years. According to Jakartaglobe (2021), Indonesia had the highest e-commerce transaction volume in Southeast Asia in 2021. During the COVID epidemic in 2021, the Indonesian government implemented a social segregation policy known as “Large-Scale Social Restrictions” (Nursalam et al., 2021). Data for this survey was collected in Indonesia during the social distancing period. To collect responses, a questionnaire was created and distributed through various online channels such as email and social media platforms like WhatsApp, Facebook, and Line.

To determine the necessary sample size for this study, the Slovin formula was employed, which uses the total population, error tolerance (5%), and minimum sample size to calculate the number of valid samples needed. The formula used was n = N / (1 + N e^2^), where n is the number of samples, e is the error tolerance, and N is the total population. The calculated minimum sample size required for this study was 286 samples. A total of 330 valid samples were collected, which is above the minimum requirement, it can be concluded that this sample size is representative of the population. This study invited a community of online shoppers in Indonesia to participate, with 1,000 invitations sent out, resulting in a response rate of 33% after screening for online shopping experience data.

To minimize sample selection bias and increase the likelihood of randomization in samples, data were primarily collected from three social media channels (Vehovar et al., 2016). To further reduce sample selection bias during data collection, a statement was included at the beginning of the questionnaire assuring participants that their answers would be anonymous and used only for this research. To ensure the absence of potential respondent biases, all samples were randomly divided into two groups and validated using the Kolmogorov-Smirnov (K-S) test (Martins et al., 2014). The K-S test results revealed no statistical difference, indicating no response bias.

### Data analysis

To analyze the data, this study used partial least squares structural equation modeling (PLS-SEM) approach, which is known to have higher item loading, average variance extracted (AVE) and composite reliability (CR) compared to the covariance based-structural equation modeling (CB-SEM) approach (Dash and Paul, 2021). The PLS-SEM approach is also suitable for explanatory analysis, which is in line with the purpose of this study (Hair et al., 2017). The researchers used the SmartPLS 3.0 software package with PLS-SEM method to estimate the mean, significance of item factor loadings, and path coefficients for the hypothesized model, to test the feasibility of measurements and hypotheses. To ensure the validity of the results, the researchers randomly divided all samples into two groups and validated them using the Kolmogorov-Smirnov (K-S) test, which indicated no response bias.

## Results

### Descriptive statistics

Table 5 illustrates that the majority of young-Millennial respondents fell within the age range of 21 to 40. Additionally, nearly 90% of the participants made at least five online purchases on a monthly basis. Furthermore, over 87% of respondents reported monthly transactions totaling more than 300,000 Indonesian Rupiah (IDR).

**Table 5:** Demographic profile.

**Socio-demographic variables**	**Description**	**Frequency**	**Percentage**
Gender	Male	136	41.2
	Female	194	58.8
Age	< 20	15	4.54
	21–30	152	46.06
	31–40	127	38.48
	>41	36	10.92
Online shopping frequency (monthly)	< 5	14	4.24
	5–10	223	67.58
	>10	93	28.18
Transaction per month	< 300 K	17	5.15
	300–600 K	142	43.03
	600–1,000 K	146	44.24
	>1,000 K	25	7.58

Table 6 presents the descriptive statistics of the measurements. The means range from 3.648 (SD = 0.761) to 4.452 (SD = 0.713) for all measurements. Among the 23 indicators, Online Shopping Continuance Intention4 (OSCI4) had the highest average value of 4.452 (SD = 0.713), while Social Contingency3 (SC3) had the lowest average value of 3.648 (SD= 0.761). In the variable of online shopping continuance intention, OSCI1 had the lowest average value of 4.073 but the highest standard deviation value of 0.787. OSCI2 had a mean value of 4.394 and a standard deviation of 0.689, while OSCI4 had a mean value of 4.452 and a standard deviation of 0.713.

**Table 6:** Descriptive statistics of measurements.

**Variable**	**Code**	**Average**	**S.D**.
Online shopping continuance intention	OSCI1	4.073	0.787
	OSCI2	4.394	0.689
	OSCI3	4.285	0.753
	OSCI4	4.452	0.713
Habit	HA1	4.158	0.632
	HA2	3.997	0.757
	HA3	4.115	0.901
	HA4	3.988	0.835
Performance confirmation	PC1	3.758	0.885
	PC2	4.200	0.736
	PC3	4.236	0.812
Effort confirmation	EC1	3.788	1.083
	EC2	4.133	0.864
	EC3	4.339	0.722
	EC4	3.927	0.794
Customer perceived value	CPV1	4.103	0.779
	CPV2	4.333	0.695
	CPV3	4.015	0.886
Social contingency	SC1	4.294	0.715
	SC2	3.767	0.811
	SC3	3.648	0.761
	SC4	3.661	0.751
	SC5	4.255	0.723

### Measurement model analysis

To ensure the reliability and validity of the variables, a measurement model was employed in this study. The measurement model test consisted of two parts: convergent validity and discriminant validity. Convergent validity was evaluated through cross-factor loading and average variance extracted (AVE). A factor loading value >0.7 and an AVE value >0.5 indicate good convergent validity for an item (Hair et al., 2014). Highly connected and positively correlated indicators within a construct with good factor loading values also indicate convergent validity (Ringle et al., 2015).

According to the results of the cross-factor loading test, two items (HA3 and EC4) had scores that were below 0.5. After removing these two items, the remaining items have cross-factor loadings above 0.7, which suggests that the construct validity is acceptable (Fornell and Larcker, 1981), as demonstrated in Table 7.

**Table 7:** Cross-factor loadings.

**Scale items**	**CPV**	**EC**	**HABIT**	**OSCI**	**PC**	**SC**
CPV1	**0.886**	0.654	0.646	0.588	0.664	0.694
CPV2	**0.888**	0.683	0.648	0.602	0.627	0.675
CPV3	**0.747**	0.567	0.464	0.413	0.489	0.518
EC1	0.637	**0.866**	0.538	0.419	0.465	0.553
EC2	0.605	**0.873**	0.570	0.488	0.489	0.548
EC3	0.740	**0.902**	0.665	0.614	0.605	0.714
HA1	0.577	0.552	**0.827**	0.525	0.487	0.602
HA2	0.490	0.469	**0.821**	0.529	0.450	0.553
HA4	0.618	0.594	**0.760**	0.557	0.503	0.609
OSCI1	0.540	0.49	0.565	**0.787**	0.488	0.562
OSCI2	0.560	0.525	0.572	**0.850**	0.478	0.579
OSCI3	0.454	0.414	0.480	**0.773**	0.403	0.492
OSCI4	0.502	0.430	0.531	**0.802**	0.507	0.491
PC1	0.513	0.441	0.460	0.466	**0.762**	0.563
PC2	0.613	0.499	0.494	0.487	**0.850**	0.576
PC3	0.619	0.523	0.523	0.492	**0.850**	0.591
SC1	0.739	0.651	0.647	0.571	0.636	**0.761**
SC2	0.408	0.406	0.453	0.452	0.494	**0.765**
SC3	0.436	0.344	0.474	0.386	0.461	**0.773**
SC4	0.412	0.332	0.471	0.373	0.430	**0.766**
SC5	0.724	0.744	0.662	0.640	0.576	**0.742**

The bold values are all factor loading values of each indicator item on its construct.

Table 8 indicates that all of the items have an AVE value that exceeds 0.5, which is a sign of acceptable convergent validity. Furthermore, the square root of the AVE value for each construct is greater than the correlations with other constructs, indicating good discriminant validity, as suggested by Hair et al. (2006).

**Table 8:** Convergent and discriminant validity.

	**AVE**	**CPV**	**EC**	**HABIT**	**OSCI**	**PC**	**SC**
CPV	0.710	**0.843**					
EC	0.775	0.755	**0.880**				
HABIT	0.645	0.704	0.675	**0.803**			
OSCI	0.646	0.643	0.582	0.671	**0.804**		
PC	0.675	0.710	0.595	0.600	0.585	**0.822**	
SC	0.580	0.753	0.694	0.735	0.663	0.701	**0.762**

The bold values are the square root value of the AVE value for each construct which is greater than the other constructs.

To test reliability, Cronbach's Alpha and composite reliability were used. Table 9 displays the composite reliability range, which was between 0.845 and 0.912, as well as the Cronbach's Alpha range, which was between 0.724 and 0.855 for the various constructs. As per the guidelines provided by Hair et al. (2012), all items had a Cronbach's Alpha value >0.7, indicating that all constructs were reliable.

**Table 9:** Composite reliability and internal consistency.

**Construct**	**Composite reliability**	**Cronbach's alpha**
OSCI	0.879	0.817
PC	0.861	0.759
EC	0.912	0.855
CPV	0.880	0.795
HABIT	0.845	0.724
SC	0.873	0.824

The goodness-of-fit (GOF) test determines whether a model adequately describes a study's empirical data (Sheppard and Meitner, 2005). To determine the GOF of a model, Tenenhaus et al. (2005) recommend using the geometric mean value of the AVE values and the average *R*^2^ value(s), as stated in Equation (1).


(1) $$\begin{array}{l} \text{GOF}=\surd \text{Average AVE }\times \text{Average }{\text{R}}^{2} \end{array}$$

The results showed GOF was 0.594, with an average AVE of 0.671 and an average *R*^2^ of 0.353. It indicated that the empirical data used in this study was suitable for measurements (Baumgartner and Homburg, 1996). The standardized root means square residual (SRMR) was used to assess the overall goodness of model fit for PLS (Hair et al., 2017). The difference between the observed correlation and the correlation matrix implied by the model is known as the SRMR. A good fit is defined as an SRMR value < 0.1 (Hu and Bentler, 1998). Results showed SRMR value was 0.098, indicating that the measurement model fits the data well.

The Normed Fit Index (NFI) was used as an indicator of global model fit in surveys with small sample sizes. NFI has a value between 0 and 1, with a higher NFI indicating a better fit (Bentler and Bonett, 1980). The chi-square goodness of fit test is estimated when comparing a sample to a population with known parameters on a specific variable. Chi-square statistics with values ranging from 0 to infinity are commonly used as goodness-of-fit indices (Wheaton, 1987). Results showed NFI = 0.709 and Chi-Square = 1293.762 indicating that the model had an acceptable fit level.

The exact model fit criteria are formed by combining two model fit indices, namely the squared Euclidean distance (d_ULS) and the geodesic distance (d_G) (Dijkstra and Henseler, 2015). Results showed that d_ULS (2.239) and d_G (0.712) had values lower than the 95% bootstrapped quantile, indicating a good fit between the measurement model and data.

### Structural model

**Figure 2:**
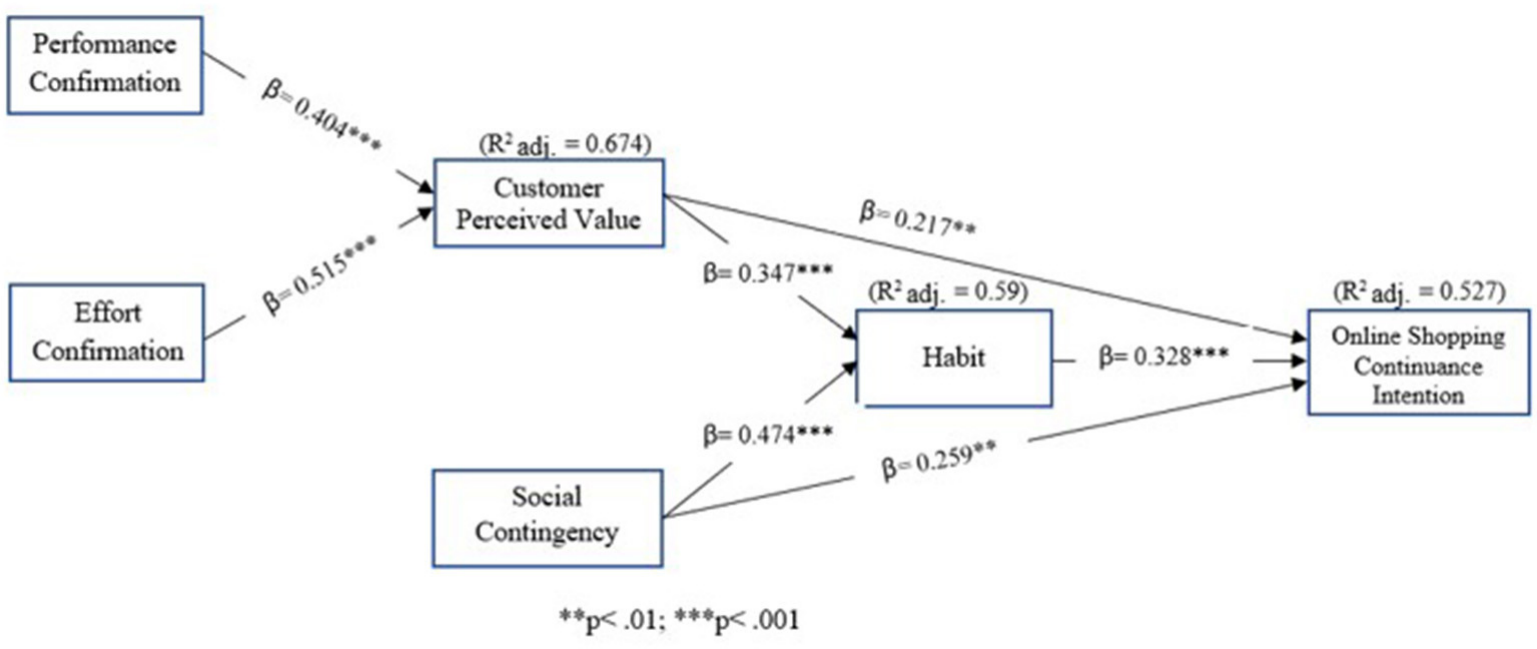
The validated model.

This study estimated the structural model using path coefficients, which indicate the strength of the relationships between independent and dependent variables (Sarstedt et al., 2017). A bootstrapping procedure was used to determine the significance of these coefficients. The adjusted *R*^2^ value, which adjusts for the number of predictors in the model (Miles, 2014), was also calculated. The results, as shown in Figure 2, revealed that all paths had positive coefficients that were statistically significant. The highest beta-value was for habit (β = 0.328^***^), followed by social contingency (β = 0.259^***^) and customer perceived value (β = 0.217^**^). Effort confirmation (β = 0.515^***^) had a greater effect on customer perceived value than performance confirmation (β= 0.404^***^). Overall, the validated model had a moderate explanatory power on consumers' online repurchase intentions, with an adjusted *R*^2^ value of 0.527. The results of the structural model supported all hypotheses, as shown in Table 10.

**Table 10:** Results of the hypotheses test.

**Path**	**Hyp**.	**β-value**	***t*-value**	***p*-value**	**Result**
PC → CPV	H1	0.404^***^	10.547	0.000	Supported
EC → CPV	H2	0.515^***^	13.599	0.000	Supported
CPV → OSCI	H3	0.217^**^	3.365	0.001	Supported
CPV → HABIT	H4	0.347^***^	6.064	0.000	Supported
SC → HABIT	H5	0.474^***^	8.75	0.000	Supported
SC → OSCI	H6	0.259^**^	3.096	0.002	Supported
HABIT → OSCI	H7	0.328^***^	4.693	0.000	Supported

^**^p-value < 0.01 and ^***^p-value < 0.001.

This study calculated effect size (ES) coefficients for each path in the validated model to compare the total effect of different variables on customers' online repurchase intentions. Table 11 presents the total ES for each path, which includes performance confirmation (ES = 0.087), effort confirmation (ES = 0.111), customer perceived value (ES = 0.330), social contingency (ES = 0.414), and habit (ES = 0.328). According to Cohen (1988), an ES of 0.35, 0.15, and 0.02 indicates a strong, moderate, and weak effect, respectively. The results indicated that social contingency had a strong total ES on online shopping continuation intention, followed by customer perceived value and habit, both of which had a moderate impact on customers' online repurchase intentions. Two external variables, performance confirmation and effort confirmation, had a weak indirect ES on online shopping retention intention.

**Table 11:** Effect sizes of variables.

**Relationships**	**Direct**	**Indirect**	**Effect size**	**Total effect**
PC → OSCI	-	0.087	0.087	Weak
EC → OSCI	-	0.111	0.111	Weak
CPV → OSCI	0.217	0.113	0.330	Moderate
SC → OSCI	0.259	0.155	0.414	Strong
HA → OSCI	0.328	-	0.328	Moderate

## Discussion and implications

### Discussion of findings

This study aimed to explore how contextual factors related to social distancing influence customers' online repurchase intentions. Empirical data was collected and analyzed to validate the research model, and the results support the hypotheses. Specifically, this study found that antecedents of customer perceived value, such as performance confirmation and effort confirmation, had a significant impact on customer perceived value. This study replaced the constructs of performance expectancy and effort expectancy from the UTAUT model with performance confirmation and effort confirmation based on ECT perspective, and this approach was found to be valid. This study showed that customers' perception of the congruence between their expectations and actual outcomes significantly influences their perceived value. Higher levels of performance confirmation and effort confirmation lead to higher perceived value.

This study's second finding was that customer perceived value had a significant impact on both habit and online shopping continuance intention. The result supported prior research (Wu et al., 2014; Pham et al., 2018; Guo and Li, 2022; Miao et al., 2022), indicating that customers are more likely to repurchase online if they received greater perceived value during their prior internet shopping experience. Moreover, this study found that customer perceived value significantly affects habit, which is consistent with prior studies (Chiu et al., 2012; Cheng et al., 2017; Nguyen et al., 2022). This result suggests that higher customer perceived value leads to more frequent habit repetition. Thus, improving customer perceived value is crucial to encourage online shopping habits and maintain customers' continued intention to shop online.

As a new factor in this study, social contingency refers to the influence of social distancing on consumer behavior, had a significant impact on habit and online shopping continuance intention. Interestingly, social contingency had the greatest effect on consumers' intention to continue online shopping compared to other variables in the model. This suggests that social contingency was a crucial aspect of online shopping during the COVID pandemic. These findings are consistent with previous research indicating that the pandemic has forced consumers to adapt their shopping habits to online channels due to social contingency (Briedis et al., 2020; Alhaimer, 2022; Kao and André L'Huillier, 2022). Therefore, it is essential for e-retailers on e-commerce platforms to consider social contingency as a contextual factor that plays a vital role in online shopping behavior, particularly during a pandemic.

Social contingency is the most significant factor influencing customers' intention to continue online shopping during the pandemic. This could be attributed to the anxiety triggered by the COVID situation, and the implementation of social distancing measures limiting people's ability to shop in physical stores. As a result, many Indonesian respondents in this study increased their online shopping, particularly for health products, due to fear of a shortage of goods resulting from panic buying. This finding builds upon previous research on Indonesian consumers (Iriani et al., 2021; Elisa et al., 2022), which demonstrated that social restrictions during the COVID pandemic have led to an increase in online sales of health products in Indonesia.

This study also found that habit has a significant impact on customers' intention to continue online shopping. This result is consistent with previous research (Khalifa and Liu, 2007; Urueña-López et al., 2012; Huang and Chen, 2017). Additionally, this research demonstrated that habit has the greatest direct influence on online shopping continuance intention compared to customer perceived value and social contingency. This finding suggests that habit is a more robust predictor of technology continuance intention, which is supported by prior research (Zhang et al., 2015; Mirkovski et al., 2018; Nguyen et al., 2022). The strong impact of habit on online shopping continuance intention could be due to consumers increasing their use of technology to shop online during unpredictable situations such as the pandemic.

This study's results suggest that the habit of online shopping among Indonesian consumers is likely to continue even after the pandemic ends. This is supported by a survey conducted by SIRCLO in 2021 (SIRCLO and Ravenry, 2021), which found that more than 40% of new Indonesian online shoppers intend to continue shopping online, indicating that online shopping has become a permanent habit during the pandemic. This finding is consistent with the current situation in Indonesia, which is one of the largest e-commerce markets globally, with an estimated increase in the number of e-commerce shoppers to 221 million IDR by 2025 (Statista, 2020).

### Theoretical implications

This study makes several distinct theoretical contributions to the existing academic literature on consumer behavior and online repurchase intention compared to previous research. Firstly, it proposes a model that considers customer perceived value (CPV) and habit as factors influencing continuous online shopping intention in the context of social distancing. Few studies have examined post-purchase consumer decisions using the UTAUT model under social distancing conditions. As such, this study establishes an influencing factors model of customers' continued online shopping intention under social distancing conditions, and modifies the UTAUT2 model based on the ECT construct to provide a more comprehensive explanation of post-purchase online consumer behavior.

Secondly, as discussed in the literature review, this study justifies the use of CPV rather than the price value construct in the UTAUT2 model. The rationale for this is that CPV has a more significant impact on repurchase intention than price value. This suggests that customers are more likely to make repeat purchases online once they have confirmed the higher value of their previous online shopping experience. Numerous studies have demonstrated the significant direct effects of CPV on online repurchase intention (Wu et al., 2014; Fang et al., 2016; Ali and Bhasin, 2019; Guo and Li, 2022; Nguyen et al., 2022). However, very few studies have explored the antecedents of CPV derived from the perspectives of ECT and UTAUT2 constructs, which significantly affect online repurchase intentions. Therefore, by drawing on theories from both the ECT and UTAUT models, this study expands on both theories by proposing a more comprehensive model.

In conclusion, this study contributes to the existing literature on online repurchase intention in three main ways that differ from related studies. Firstly, it proposes a model based on CPV and habit for continuous online shopping intention under the context of social distancing, which is an area that has not been explored in previous studies. Secondly, it argues for the use of CPV instead of price value in the UTAUT2 model, as CPV has a greater impact on repurchase intention. Finally, this study introduces social contingency as a new variable that affects online repurchase decisions and suggests that it can be applied to other social occurrences in future research. By addressing these gaps in the literature, this study provides a foundation on post-purchase behavior in online consumer decision-making.

### Managerial implications

To retain customers in the post-COVID era, online retailers should take note of the practical implications from this study. The results suggest that improving performance confirmation and effort confirmation can significantly affect customer perceived value. This can be achieved by enhancing the speed of online order delivery, simplifying the ordering process, streamlining payment processing, and improving overall convenience and practicability of the online shopping experience. Furthermore, providing excellent customer service can help online retailers better understand their customers and strengthen their perceived value. These measures can increase customer satisfaction and lead to higher repurchase intentions, ultimately benefiting online retailers in the long run.

The results of this study indicate that customer perceived value (CPV) plays a crucial role in online shopping habits and repurchase intentions in the context of e-commerce. The findings suggest that customers are more likely to make repeat purchases online when they perceive higher value for money and desired benefits. Therefore, online retailers should focus on providing good value for money and offer various promotions and discounts to encourage customers to develop online shopping habits and make repeat purchases in the future.

Social contingency was found to have the largest effect on consumers' online shopping continuance intention compared to all other variables in this study. Hence, online retailers should prioritize ensuring the availability of necessities such as medicine, masks, and other health products during social distancing in the pandemic. Online retailer managers could encourage the regular use of their platforms by offering incentives such as promotional items like masks, hand antiseptic, or disinfectant. They could also offer free protective suits for COVID to reinforce their brand image and increase customer loyalty.

This study revealed that habit is a significant factor in online shopping continuance intention. It was found that habit has a stronger direct effect on customers' online shopping intentions compared to perceived value and social contingency. As a result, online retailers can implement loyalty programs that incentivize repeat purchases and customer retention. This can include reward points, discounts, and exclusive offers for loyal customers, which can help reshape their online shopping habits and encourage repurchase intention. Additionally, online retailers should focus on providing a seamless and personalized shopping experience to enhance customer satisfaction and loyalty. Online retailers can use endorsers or influencers to promote their products through live-stream shopping to attract, interact and maintain engagement with customers to stimulate customers to make repeat online purchases.

### Limitations and future recommendations

To enhance the model's applicability, several limitations need addressing in future research. Firstly, it is necessary to validate the generalizability of the findings to other countries outside Indonesia. Future studies should investigate the proposed model's applicability to other Asian countries. Secondly, this study's cross-sectional data sampling during the COVID pandemic makes it challenging to understand the effects over time. Thus, future research should incorporate longitudinal data to examine the differences in the pre-COVID, COVID, and post-COVID periods. Finally, to gain a better understanding of online repurchase behaviors, future studies may consider including moderating variables.

## Data availability statement

The original contributions presented in the study are included in the article/supplementary material, further inquiries can be directed to the corresponding author.

## Author contributions

WW and GW conceptualized the study. WW contributed to the study design, reviewed, edited, and managed the manuscript. GW contributed to the study design, collected and analyzed the data, and wrote the original draft. DR reviewed and edited the manuscript.
